# Repetitively burst-spiking neurons in reeler mice show conserved but also highly variable morphological features of layer Vb-fated “thick-tufted” pyramidal cells

**DOI:** 10.3389/fnana.2022.1000107

**Published:** 2022-10-28

**Authors:** Jochen F. Staiger, Alexandra Sachkova, Martin Möck, Julien Guy, Mirko Witte

**Affiliations:** Institute for Neuroanatomy, Universitätsmedizin Göttingen, Georg-August-Universität Göttingen, Göttingen, Germany

**Keywords:** somatosensory (barrel) cortex, layer Vb-fated pyramidal cells, whole cell patch clamp recording, action potential firing pattern, biocytin filling, neuronal reconstructions, reeler mutant

## Abstract

Reelin is a large extracellular glycoprotein that is secreted by Cajal-Retzius cells during embryonic development to regulate neuronal migration and cell proliferation but it also seems to regulate ion channel distribution and synaptic vesicle release properties of excitatory neurons well into adulthood. Mouse mutants with a compromised reelin signaling cascade show a highly disorganized neocortex but the basic connectional features of the displaced excitatory principal cells seem to be relatively intact. Very little is known, however, about the intrinsic electrophysiological and morphological properties of individual cells in the reeler cortex. Repetitive burst-spiking (RB) is a unique property of large, thick-tufted pyramidal cells of wild-type layer Vb exclusively, which project to several subcortical targets. In addition, they are known to possess sparse but far-reaching intracortical recurrent collaterals. Here, we compared the electrophysiological properties and morphological features of neurons in the reeler primary somatosensory cortex with those of wild-type controls. Whereas in wild-type mice, RB pyramidal cells were only detected in layer Vb, and the vast majority of reeler RB pyramidal cells were found in the superficial third of the cortical depth. There were no obvious differences in the intrinsic electrophysiological properties and basic morphological features (such as soma size or the number of dendrites) were also well preserved. However, the spatial orientation of the entire dendritic tree was highly variable in the reeler neocortex, whereas it was completely stereotyped in wild-type mice. It seems that basic quantitative features of layer Vb-fated RB pyramidal cells are well conserved in the highly disorganized mutant neocortex, whereas qualitative morphological features vary, possibly to properly orient toward the appropriate input pathways, which are known to show an atypical oblique path through the reeler cortex. The oblique dendritic orientation thus presumably reflects a re-orientation of dendritic input domains toward spatially highly disorganized afferent projections.

## Introduction

The reeler mouse has served the developmental neuroscience community as an insightful model system to begin understanding the seminal contributions made by reelin-secreting Cajal-Retzius cells during cortical development (Caviness et al., 1988; D'Arcangelo et al., 1997; Tissir and Goffinet, 2003; Zhao and Frotscher, 2010; Prume et al., 2018). A strong notion from early on was that the reeler cortex is inverted but preserves correct long-range wiring with different subcortical areas or nuclei (Caviness and Sidman, 1973; Caviness and Frost, 1983; Yoshihara et al., 2010; Imai et al., 2012). Another milestone was the discovery of the reelin receptors and the associated intracellular signaling mechanisms (Trommsdorff et al., 1999; Bock et al., 2003; Herz and Chen, 2006) that, when disturbed, also result in a reeler-like phenotype of laminated brain structures. This has guided researchers to understand the different reelin-dependent steps of neuronal migration and differentiation that lead to a mature six-layered neocortex (Polleux et al., 1998; Hack et al., 2007; Valiente and Marin, 2010; Franco et al., 2011; Klingler et al., 2021). However, the validity of the inversion hypothesis, on which most concepts of reelin function do rest, has subsequently been questioned (Dekimoto et al., 2010; Wagener et al., 2010; Boyle et al., 2011). The consensus reached in these papers is that in different cortical areas, different patterns of disorganization can be found, with the largest degree of cellular dystopia being found in the primary somatosensory (barrel) cortex.

Interestingly, in the reeler barrel cortex, sensory representation is rather well preserved and columnar modules respond well to artificial or more natural, behaviorally relevant whisker stimulation (Guy et al., 2015; Wagener et al., 2016). Furthermore, such a largely intact function was also shown in the visual cortex where fine-grained retinotopic maps were detected [in general agreement with Drager (1981); Simmons and Pearlman (1982)] that went along with remarkable visually guided behavioral performance and increased ocular-dominance plasticity (Pielecka-Fortuna et al., 2015). This leads to the interesting question of to what extent the individual neurons making up the circuits for sensory information processing and representation are altered by a lack of reelin during the entire cortical development, leading to a cortex without layers (Guy and Staiger, 2017). It is of interest to note that we and others have found that the number of layer-fated neurons is comparable for LIV- and LV-neurons, whereas it seems that late-born LII/III neurons are more numerous in the reeler cortex (Polleux et al., 1998; Wagener et al., 2010).

So far, very few studies of the reeler cortex focused on the properties of single neurons. A few Golgi staining reports exist, which show that pyramidal cells have atypical orientations of the dendritic tree and axon initial segments from early development on into late adulthood (Pinto Lord and Caviness, 1979; Landrieu and Goffinet, 1981). However, given the now-established fact that pyramidal neurons in each layer possess a unique feature set (Molyneaux et al., 2007; Huang, 2014; Narayanan et al., 2017), it is not clear which types of pyramidal cells actually show these morphological alterations in Golgi stains. Similarly, the only single-cell intracellular electrophysiological characterization of reeler pyramidal cells presented evidence that the general intrinsic and extrinsic neurophysiological properties, including regular-spiking and intrinsically burst-spiking action potential firing patterns, are unaltered in the neocortex (Silva et al., 1991). Since this latter study provided only a much reduced qualitative description of the somatodendritic domain of the recorded neurons and did not include axonal reconstructions, it remained unclear how well the correlation of structure with function (Schubert et al., 2001; Feldmeyer, 2012; Staiger and Petersen, 2021) is preserved for any of the cortical pyramidal cell types in reeler mice.

Therefore, with the present study, we set out to re-examine this question for the only type of pyramidal cell in the reeler cortex that can be compared to wild-type neurons without the use of genetic labeling techniques, namely the repetitively burst-spiking (RB) pyramidal cells, which exclusively populate layer Vb in the wild type neocortex (Chagnac-Amitai et al., 1990; Larkman and Mason, 1990; Hattox and Nelson, 2007; Staiger et al., 2016). We first characterized the electrophysiological properties of these neurons, while filling them with biocytin in acute slice preparations of young adult wild-type and reeler mice. This label was visualized with silver-intensified DAB staining, which allowed us to three-dimensionally and quantitatively reconstruct the somatodendritic compartment of these neurons, as well as qualitatively the local recurrent collaterals of the axonal arbor, which was retained in the slice. The main findings are as follows: (i) reeler RB pyramidal cells were mostly found in the superficial third of the cortical depth, where they showed physiological and morphological properties that were quantitatively not significantly different from wild-type controls; and (ii) qualitatively, reeler RB cells displayed any orientation of the apical dendrite, ranging from “upright” *via* oblique to inverted, whereas the recurrent axonal collaterals were closer to an “inverted” pattern, with horizontal branches mainly ramifying at the level of the soma location.

## Materials and methods

### Slice preparation

All experiments were performed in accordance with German law on the Protection of Animals. Male and female mice [*n* = 30; postnatal days 20-32, consisting of 12 B6C3Fe wild-type and 18 B6C3Fe rl^−^/^−^ mice (reeler)] were deeply anesthetized with isoflurane and decapitated. Thalamocortical slices of 300 μm thickness, containing the primary somatosensory cortex barrel cortex; (Porter et al., 2001), were produced by vibratome sectioning (VT1200S, Leica; Germany). The cold (4°C) cutting solution contained (in mM) 75 sucrose, 87 NaCl, 2.5 KCl, 0.5 CaCl_2_, 7.0 MgCl_2_, 26 NaHCO_3_, 1.25 NaH_2_PO_4_, and 10 glucose, continuously equilibrated with 95% O_2_ and 5% CO_2_, pH 7.4. Slices were incubated for 0.5 to 1 h at 32°C before recording in extracellular solution [artificial cerebrospinal fluid (ACSF)] of the following composition (in mM): 125 NaCl, 2.5 KCl, 2 CaCl_2_, 1 MgCl_2_, 26 NaHCO_3_, 1.25 NaH_2_PO_4_, and 25 glucose, pH 7.4, when equilibrated with 95% O_2_ and 5% CO_2_.

### Electrophysiological recordings

Slices were transferred to a fixed-stage recording submerged chamber (standard ACSF flow rate of 1–2 ml/min at 32°C) in an upright microscope (Axioskop FS; Carl Zeiss, Germany). The barrel field was visualized at low magnification (2.5x) under brightfield conditions and large pyramidal cells (i) in layer Vb in vertical register with a layer 4 barrel (for wild type mice) or (ii) on top of cellular aggregates resembling “barrel equivalents” (for reeler mice) were selected using a 40 × water immersion objective (40 × /0.75 W; Olympus, Germany) under infrared-enhanced quarterfield illumination. For whole-cell patch-clamp recordings, filamented borosilicate glass capillaries (Science Products, Hofheim, Germany) of 5 to 8 MΩ resistances were filled with (in mM) 117 K-gluconate, 13 KCl, 10 K-HEPES, 11 EGTA, 2 Na_2_ATP, 0.5 NaGTP, 1 CaCl_2_, 2 MgCl_2_, 11 EGTA, and 0.5% biocytin. Membrane potentials were recorded using an SEC-05L amplifier (npi electronics, Tamm, Germany) in discontinuous current-clamp mode with a switching frequency of 50 kHz, filtered at 3 kHz, and digitized at 10 to 25 kHz using an LIH-1600 interface (Heka Elektronik, Germany). Access resistance was monitored and compensated if changes appeared. Recordings during which the access resistance could not be compensated were discarded. Data were recorded and stored with PC-based software (TIDA 5.2 for Windows; Heka Elektronik, Germany). Analyses of electrophysiological data were done with custom-made programs written in Signal5 script language (Cambridge Electronic Design, Cambridge, UK). Data were not corrected for an estimated liquid junction potential of 10 mV. Passive and active properties of a neuron were determined immediately after reaching whole-cell configuration by applying 1 s long hyperpolarizing (−50 pA) or depolarizing (10 to 300 pA) rectangular current pulses of varying strength at resting membrane potential. We classified the recorded cells using their firing properties into regular or burst-spiking pyramidal neurons.

### Analysis and statistics

The resting membrane potential (V_m_) was calculated for each trace by averaging all data points before current pulse applications. The input resistance (R_in_), the membrane time constant (τ), and the membrane capacitance (C_m_) were determined from averages of membrane potential responses to 5 to 10 consecutive square current pulses (−50 pA, 1 s duration). R_in_ was calculated according to Ohm's law, τ was determined by fitting an exponential [f(x) = ae – x/b +c; a: amplitude, b: time constant, c: most negative membrane potential value]. Because h-current was present in most of the cells, R_in_ and τ were determined for the maximal voltage response, that is, prior to h-current activation. C_m_ was calculated according to C_m_ = τ/R_in_. Additionally, we also calculated the steady-state input resistance (ssR_in_) for the last 20 ms of the voltage response before the current pulse was turned off. This allowed us to estimate the strength of the h-current by calculating a sag index: sag index (%) = [(1/ ssR_in_- 1/R_in_)/ 1/ssR_in_] × 100. Rheobase was measured by increasing the amplitude of clearly subthreshold square current pulses (1 s duration) by 5 pA each step until the firing threshold was reached. The firing threshold was estimated by a backward search for the extrapolated membrane potential value corresponding to 10% of the slope of the upstroke. Action potential (AP) amplitude was calculated as the difference between the firing threshold and AP peak. AP width is the duration of the AP at 50% of the amplitude. The maximal slope of APs is given by the maximum found in the first derivative of the upstroke. All measurements of AP properties were done using rheobase stimulations and only for the first two AP of a burst. Inter-spike intervals were determined by calculating the period between the peaks of the first and the second AP in a burst. The recorded busting cells displayed a monophasic medium afterhyperpolarization (mAHP). The amplitude of afterhyperpolarizations was determined by measuring the difference in voltage from the firing threshold to the maximum deflection of the repolarization. The peak time of AHP was measured from the time point the repolarization of the action potential crossed the firing threshold to the maximum amplitude of the AHP.

For statistical comparisons (Sigma Plot 13, Systat Software Inc., USA), data were tested for normality (Shapiro–Wilk test) and equal variance. If both passed, a one-way Student's *t*-test was used. If one or both failed, a Mann–Whitney rank sum test was used. All values are given as mean ± SD.

### Histological procedures

After recording, slices were fixed in phosphate-buffered 4% paraformaldehyde for 24 h at 4°C. For visualization of the biocytin-filled neurons, slices were processed as described previously (Staiger et al., 2015). The barrel field was either visualized by cytochrome oxidase histochemistry or the barrel pattern of the micrograph of the native slice was transferred manually into the reconstruction. Reconstruction and morphological analysis of the biocytin-labeled neurons were performed using a Nikon Eclipse 80i (Nikon, Germany) attached to a computer system (Neurolucida; MBF Bioscience Europe). Data were not corrected for tissue shrinkage (which was estimated to be ~15% in x-/y-dimensions and ~40–50% in z-dimension). The reconstructed cells were quantitatively analyzed with Neurolucida Explorer (MBF Bioscience Europe).

### Statistical analysis

Statistical analysis of individual parameters was performed using two-tailed Student's *t*-tests and *post-hoc* Bonferroni correction (SigmaPlot 14; Systat Software Inc.). If not mentioned differently, data are presented as mean ± SEM.

## Results

### Electrophysiological characterization of repetitively burst-spiking pyramidal cells

Only neurons with an intrinsic burst-spiking ability at rheobase stimulation and with a stable resting membrane potential of −55 mV were included in the analysis, which led to a sample of 11 wild-type and 17 reeler neurons (Table 1). In both genotypes, neurons showed comparable, statistically and not significantly different passive membrane properties, like, for example, input resistance (WT: 72.6 ± 17.3 ms; rl-/-: 76.8 ± 19.9 ms) or membrane time constant (WT: 11.3 ± 3.1 ms; rl-/- 10.1 ± 3.3 ms), which is in general agreement with previous studies (Silva et al., 1991; Hattox and Nelson, 2007). A sag index of 30.2 ± 7.4% in WT vs. 26 ± 7.9% in rl-/- suggests a comparable incidence of H-current. Moreover, active membrane properties did not differ between the genotypes, with one exception, namely the first inter-spike interval, which was 5.5 ± 0.7 ms for WT vs. 4.8 ± 0.8 ms for rl-/- (*p* = 0.011). Whether this subtle difference acquires physiological meaning in the target cells remains a matter of further experiments.

**Table 1:** Basic subthreshold and suprathreshold electrophysiological properties of RB pyramidal cells of WT and reeler mice.

	**WT (*n =* 11)**	**reeler (*n =* 17)**	***p*** **value**
**postnatal age [days]**	24.6 ± 3.3	25.5 ± 3.7	*p =* 0.232
**V**_**m**_ **[mV]**	−59.1 ± 3.1	−59.9 ± 3.3	*p =* 0.518
**R**_**in**_ **[MΩ]**	72.6 ± 17.3	76.8 ± 19.9	*p =* 0.569
**τ [ms]**	11.3 ± 3.1	10.1 ± 3.3	*p =* 0.328
**C**_**m**_ **[pF]**	161.8 ± 49.1	138.2 ± 52.7	*p =* 0.246
**sag index [%]**	30.2 ± 7.4	26 ± 7.9	*p =* 0.165
**rheobase [pA]**	74.3 ± 46.2	94.4 ± 42.1	*p =* 0.245
**1. AP threshold [mV]**	−43 ± 3.1	−43.1 ± 3.5	*p =* 0.965
**1. AP amplitude [mV]**	81.5 ± 9.2	79.6 ± 9.8	*p =* 0.609
**1. AP width [ms]**	0.9 ± 0.1	0.9 ± 0.2	*p =* 0.522
1. AP max slope [V*s]	256 ± 51	265 ± 80.8	*p =* 0.742
**2. AP threshold [mV]**	−40.6 ± 3.7	−40.3 ± 3.4	*p =* 0.850
**2. AP amplitude [mV]**	71.1 ± 7.0	68.9 ± 9.5	*p =* 0.513
**2. AP width [ms]**	1.1 ± 0.2	1.1 ± 0.2	*p =* 0.584
2. AP max slope [V*s]	206.3 ± 45.1	212.4 ± 65.9	*p =* 0.791
**mAHP amplitude [mV]**	−18.8 ± 2.5	−18.8 ± 2.1	*p =* 0.965
**mAHP time to peak [ms]**	71.9 ± 19.9	57.5 ± 17.1	*p =* 0.052
**1. ISI [ms]**	5.5 ± 0.7	4.8 ± 0.8	*p =* 0.011*

Values are given as mean ± standard deviation.

**Figure 1:**
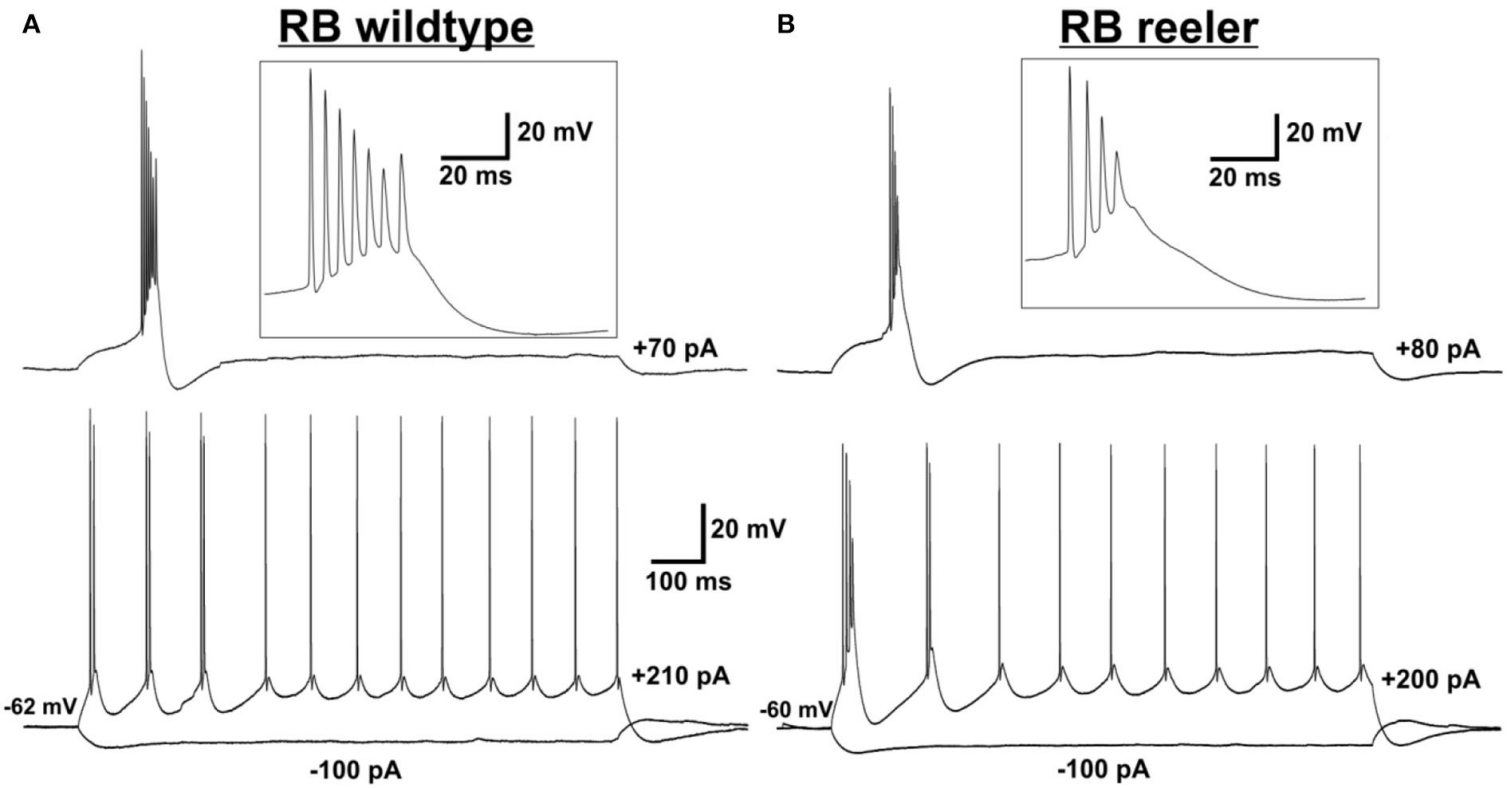
Electrophysiological characterization of (LVb) repetitively burst-spiking (RB) pyramidal cells. **(A,B)** Representative examples for RB pyramidal cells in LVb of WT **(A)** and reeler **(B)** mice. Voltage responses to rheobase stimulation (upper traces) and to depolarizing suprathreshold and hyperpolarizing stimulation (lower traces). Inserts show the burst at rheobase at higher temporal resolution. **(A)** Example RB cell of WT mouse firing one burst (with up to 7 APs) at rheobase level and three bursts and several single APs in response to suprathreshold stimulation. Also, a sag is visible after a hyperpolarizing (−100 pA) stimulus. **(B)** Example RB cell of reeler mouse firing one burst (with up to 4 APs) at rheobase level and two bursts and several single APs in response to suprathreshold stimulation. A sag is visible after a hyperpolarizing (-100 pA) stimulus.

The action potential firing pattern, by default, was classified as repetitively burst-spiking (Figure 1), with our definition of a burst being 2 or more closely spaced action potentials riding on a depolarizing envelope at rheobase (i.e., threshold) stimulation strength. Furthermore, *repetitive* burst-spiking implies that at suprathreshold stimulation at least two episodes of bursting had to occur before the firing pattern switched to individual action potentials. Thus, we do not present here the numerous regular-spiking or initial doublet-spiking pyramidal neurons that were recorded as well (cf. Silva et al., 1991; Staiger et al., 2016). The reason for this selection is that, although the latter firing patterns can be related to specific cell types *via* their laminar location in wild-type animals, this is impossible in the reeler neocortex. The present procedure allowed us to compare two similar if not identical types of neurons between the two genotypes.

### Morphological characterization of repetitively burst-spiking pyramidal cells

**Figure 2:**
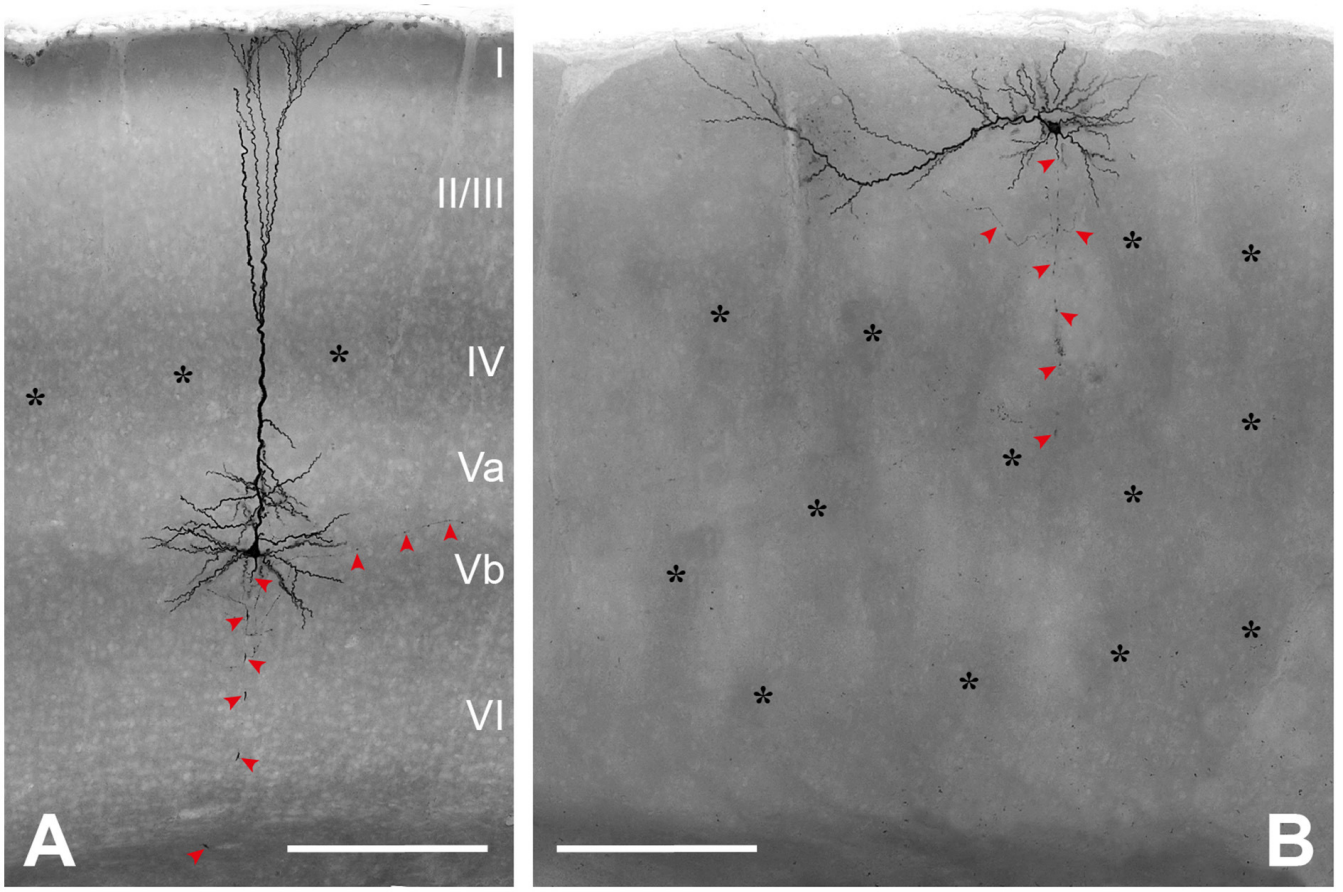
Morphological characterization of (LVb) repetitively burst-spiking (RB) pyramidal cells. **(A)** Representative example of an RB pyramidal cell in LVb of WT. **(B)** Representative example of an RB pyramidal cell close to the pial surface and on top of barrel equivalents in reeler. Both images represent minimum intensity projections of all-optical planes containing biocytin-labeled profiles, which mask details of the axonal arbor (red arrowheads). Asterisks mark barrels **(A)** or barrel equivalents **(B)** see also Wagener et al. (2016). Scale bars: 250 μm.

Only neurons that were located at least 50 μm below the slice surface and were oriented with their apical dendrite perpendicularly to the cutting angle were included for morphological analysis. Nevertheless, in all these neurons, dendritic and axonal profiles of higher order were severed at the slice surfaces, because of the extensive nature of the respective trees (Figure 2). Due to the arbitrary orientation of the reeler RB pyramidal cells, with their apical dendrites pointing into any possible direction (also in the z-plane of the slice), the sample includes only 5 reeler vs. 13 wild-type neurons that were reconstructed with Neurolucida. Altogether, as for the electrophysiological properties, the similarities of features outweigh strongly the differences between reeler and wild-type neurons and, therefore, no statistically significant parameters were detected (Table 2).

**Table 2:** Somatic and dendritic morphological properties of RB pyramidal cells of WT and reeler mice.

	**WT (*n =* 13)**	**reeler (*n =* 5)**	***P*** **value**
**Feret max (μm)**	21.34 ± 2.47	20.96 ± 1.99	*p =* 0.794
**Feret min (μm)**	15.29 ± 1,69	14.08 ± 1.07	*p =* 0.157
**Soma (μm** ^**2**^ **)**	242.34 ± 55.55	212.86 ± 23.23	*p =* 0.266
**Number of dendrites**	6.61 ± 0.78	8 ± 1.82	*p =* 0.128
**Total dendritic length (μm)**	9,510 ± 1,688	8,883 ± 331	*p =* 0.544
**Apical dendritic length (μm)**	6,679 ± 1,611	5,063 ± 1,691	*p =* 0.133
**Basal dendritic length (μm)**	2,831 ± 794	3,821 ± 1,830	*p =* 0.179
**Total number of endings**	69 ± 9	73 ± 6	*p =* 0.805
**Apical ends**	44 ± 9	37 ± 14	*p =* 0.303
**Basal ends**	25 ± 5	34 ± 10	*p =* 0.406
**Apical max diameter (μm)**	5.31 ± 1.62	5.50 ± 1.59	*p =* 0.834
**Basal max diameter (μm)**	2.53 ± 0.57	3.32 ± 0.55	*p =* 0.226
**Basal min diameter (μm)**	0.7 ± 0.22	0.64 ± 0.05	*p =* 0.580

Values are given as mean ± standard deviation. max – maximum, min – minimum.

### Somatodendritic properties

Soma size was comparable for both genotypes, with the “height” (feret max) being 21.34 ± 2.47 μm in the wild-type vs. 20.96 ± 1.99 μm in rl-/- (*p* = 0.794) and the “width” 15.29 ± 1.69 μm (WT) vs. 14.08 ± 1.07 μm (rl-/-; *p* = 0.157). Soma shape is strongly dictated by the dendritic configuration. Since the dendritic tree, containing one apical dendrite and mostly four to seven basal dendrites, showed a clear polarization in the wild-type, which was to a degree maintained in reeler, in both genotypes somata displayed an ovoid to pyramidal shape.

A total dendritic length of 9,510 ± 1,688 μm in WT vs. 8,883 ± 331 μm in rl-/-, with the total number of endings amounting to 69 ± 9 in WT vs. 73 ± 6 in rl-/-, speaks in favor of a substantial recovery of the dendritic trees of these largest neurons of the rodent cerebral cortex. Furthermore, the largest diameter of the primary dendrites (at their origin from the soma) showed comparable values for the apical (WT: 5.31 ± 1.62 μm; rl-/-: 5.50 ± 1.59 μm) as well as the basal dendrites (WT: 2.53 ± 0.57 μm; rl-/-: 3.32 ± 0.55 μm).

### Axonal properties

Since all axons were obviously truncated (in reeler mice more strongly than in wild-type animals), and in the mutant, the laminar reference space is missing, the description of axonal properties is kept at a qualitative level.

**Figure 3:**
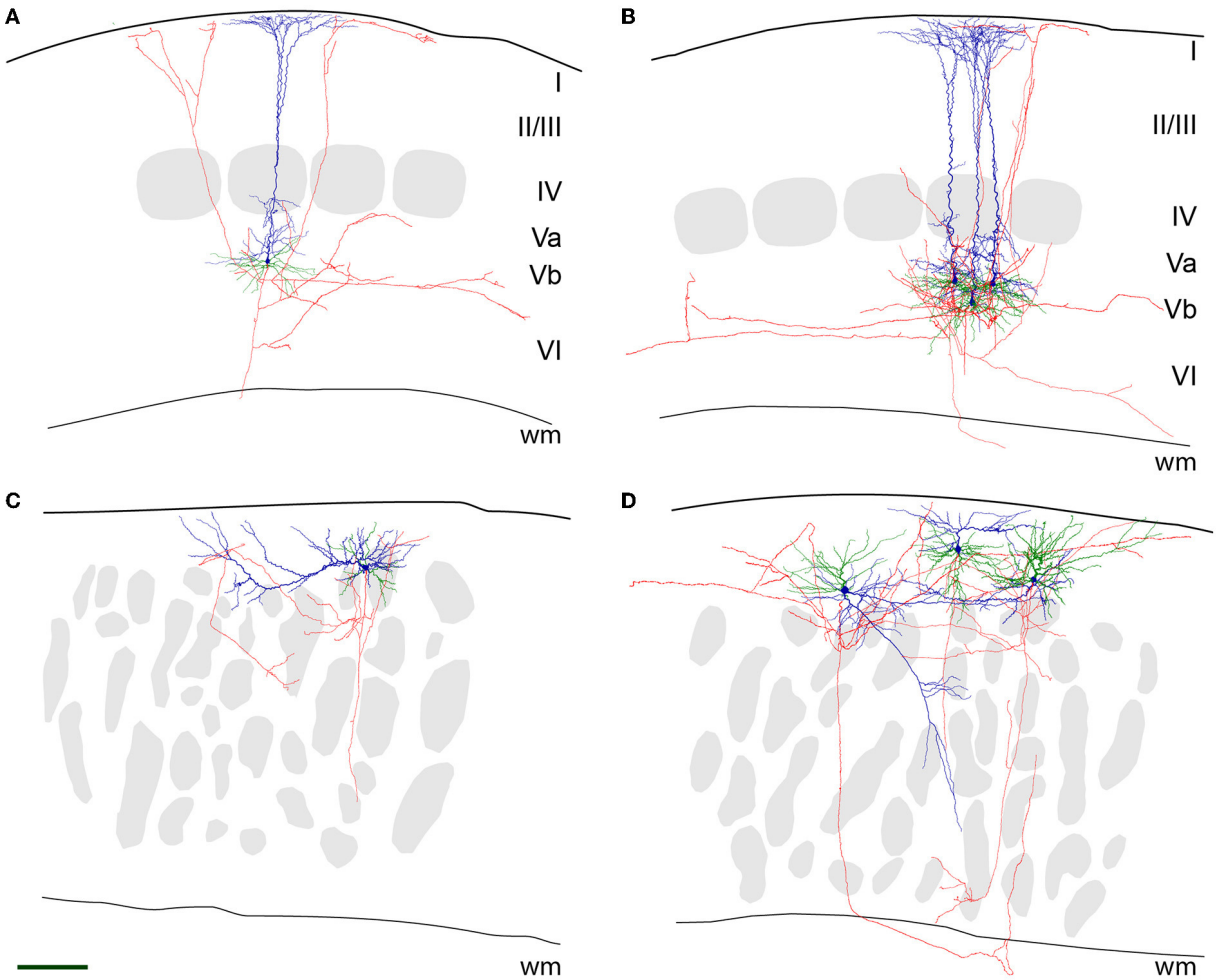
Neurolucida reconstructions of (LVb) repetitively burst-spiking (RB) pyramidal cells. Representative examples for RB pyramidal cells in LVb of WT **(A,B)** and close to the pial surface in reeler **(C,D)** mice. The pial surface and white matter (wm) border are delineated by a thicker and a thinner black line, respectively. Soma and apical dendrite is represented in blue, basal dendrite in green, and axon in red. Gray patches in **(A,B)** are barrels, and in **(C,D)** barrel equivalents (see also Figure 2). Scale bar: 150 μm. **(A)** A single RB cell and **(B)** three superimposed RB cells of WT mice are shown. As is typical for these cells, the large soma emits an apical dendrite with oblique side branches toward the pial surface, which starts to form a terminal tuft as early as in LIV. Basal dendrites originate at the opposite pole of the soma and spread out (semi-)radially. The axon runs toward the white matter and gives off recurrent collaterals that either ascend to LI or prefer a horizontal to an oblique direction in the infragranular layers. **(C)** A single RB cell and **(D)** three superimposed RB cells of reeler mice are shown. Note that in **(C)**, the apical dendrite has a horizontal whereas in **(D)**, from left to right) an oblique, another horizontal and an “upright” orientation can be seen. The segregation from the basal dendrites is much less clear. Note that the axons by and large prefer horizontal directions in the compartment being located between the pial surface and the barrel equivalents.

In wild-type LVb RB pyramidal cells, the axon originated opposite to the apical dendrite at the base of the soma and ran toward the white matter. Either in L6 or the white matter, the axon was lost at the slice surface, however, not without emitting numerous collaterals during this trajectory. These collaterals took up a recurrent and slightly oblique ascending course, reaching up to LI, where they formed horizontally elongated patches of terminal arborization (Figures 3A,B). In addition, on a regular basis, horizontally oriented collaterals were also traced, extending over several neighboring barrel territories. Some branches always ramified in LVI as well, whereas LIV was usually passed without the formation of local collaterals.

A similar, however, “close-to-inverted” pattern, with some additional features, can be described for reeler RB cells. Although in all cases, the axon initial segment was directed toward the white matter as well, its precise point of origin was very variable, ranging from a conventional somatic origin to dendritic origins (with the dendrite either allocated to the apical or basal category). This descending main stem gave off collaterals that showed a preferential horizontal distribution, which showed the highest density at a superficial location, namely between the pial surface and the top of the barrel equivalent covering zone (Figures 3C,D). In addition to the local and the horizontal collaterals, irregular (quasi-circular) trajectories were observed. In the left example of Figure 3D, the axon first exits the cortical gray matter, only to re-enter and deeply penetrate it.

## Discussion

With the present study, we achieved a better characterization of a key cell type in the neocortex of reeler mouse mutants and wild-type controls, namely the layer Vb-fated “thick-tufted” pyramidal cell (Ramaswamy and Markram, 2015; Staiger et al., 2016; Narayanan et al., 2017). These long-range projecting neurons are now often called “extratelecephalic” or “pyramidal-tract” neurons due to their exclusive connectivity profile, making them the only cortical neuron type that is capable of transferring highly integrated sensorimotor output to motor command centers throughout the entire neuraxis (Harris and Shepherd, 2015; Economo et al., 2018; Staiger and Petersen, 2021). This function is probably supported by their also unique action potential firing pattern, being repetitively burst-spiking, which should be an effective means for a “safe transmission” *via* synapses onto their target neurons (Lisman, 1997; Li et al., 2009; Berger et al., 2010). Here, we have analyzed some morphoelectrical properties of these neurons in slices of the reeler barrel cortex, a fascinating “experiment of nature” to supply us with a model system in which to test for putative functions of cortical layers in microcircuitry organization (Guy and Staiger, 2017) in a condition also relevant for the human brain (Romero et al., 2018). Altogether, our results show remarkable preservation of intrinsic electrophysiological and somatodendritic morphological properties, while axonal arbors display unusual trajectories that can be considered as an adaptation to spatially highly variable dendritic tree orientations.

### Methodological considerations

Acute slice experiments continue to deliver valuable data on cortical cell types and their varied properties (cf. Jiang et al., 2015; Gouwens et al., 2019). However, especially for the large pyramidal cells, both the dendritic tree and the axonal arbor will be truncated to a variable extent. Whereas the dendrites of the largest cortical neurons (i.e., the layer Vb thick-tufted pyramidal cells) could, in optimal cases, obtain a satisfactory recovery ratio (of an estimated up to 85% completeness), calculations were performed on the axon point toward a small recovered fraction (Stepanyants et al., 2009; Narayanan et al., 2015). However, in these estimates, the entire axon with all subcortical targets was partly taken into consideration. When one focuses on the intracortical recurrent collaterals, at least the qualitative pattern between *in vitro* and *in vivo* studies has turned out to be comparable (Stepanyants et al., 2008; Narayanan et al., 2015). In terms of electrophysiological properties, no major differences in intrinsic physiology between *in vitro* and *in vivo* studies has been found (Connors et al., 1982; Zhu and Connors, 1999; Gentet et al., 2010).

Another point worth mentioning is the developmental maturation of the burst-spiking behavior and the dendritic tree. As shown in rats, a multitude of functional and structural features change during early postnatal life (cf. Markram et al., 1997; Zhu, 2000). In a conclusive study from Kasper et al. (1994), it is stated: “It was not possible to elicit such bursts from any neurons before P15; by P21 all “thick/tufted” cells recorded had become bursters.” Since the same study also reported that “from P5 on, the apical dendrites of neurons could easily be classified as “thick/tufted” or “slender/untufted”, ” we feel safe to conclude that our sample was not affected by developmental processes leading to a false classification of neurons.

### Intrinsic physiology of RB pyramidal cells

A prevailing hypothesis derived from studies with developmental mouse mutants or cell culture systems was that most morphological but also physiological single-cell properties are determined at the moment of cell birth (Caviness et al., 1988; Tissir and Goffinet, 2003; Linaro et al., 2019; Molnar et al., 2020). Thus, it is not surprising that the few already existing physiological studies on cortical reeler neurons have not described any peculiar abnormality or special feature but largely could reproduce the standard physiology of the wild-type control animals (Silva et al., 1991; Kowalski et al., 2010; Guy et al., 2016). The same can be said about similar mouse models, like Lis1-mutant mice (D'Amour et al., 2020). So, in agreement with Silva et al. (1991), we here show that virtually all passive and active electrophysiological properties between reeler and wild-type LVb-fated pyramidal neurons are statistically not significantly different. The only exception being the first interspike interval, which was slightly shorter in reeler neurons, still needs to be examined for functional significance.

It is of interest to note that the sag index we measured speaks in favor of a relatively strong I_h_, at least in those dendritic compartments that can be accessed *via* somatic whole-cell recordings. In a study by Kupferman et al. (2014), it was shown that Dab1-mutant CA1 pyramidal cells display strongly impaired synaptic integration in the distal apical dendrite due to a defective gradient of HCN channels caused by disturbed reelin signaling. In future studies, it will be interesting to determine whether this applies to reeler LVb-fated RB cells as well. This is but one indication that domain-specific changes in dendritic excitability might exist between LVb neurons in WT and reeler mice.

### Morphological properties of RB pyramidal cells

Although for rat cortex there are numerous studies of LV pyramidal cells both, *in vitro* and *in vivo* [reviewed in Ramaswamy and Markram (2015)], we do not know of a quantitative morphological description of this cell type in the mouse. Thus, it is currently impossible to discuss our data with regard to previous publications. However, generally, our wild-type control data are in agreement with Hattox and Nelson, who studied “corticotrigeminal pyramidal cells,” which belong to the bursting type (Hattox and Nelson, 2007).

For the reeler neurons, previous studies presented some qualitative dendritic reconstructions, which however seem to be very incomplete (Landrieu and Goffinet, 1981; Silva et al., 1991). Here, we want to emphasize that we consider the reeler and wild-type neurons as quantitatively identical (dendritic length, diameter, number of branches, etc.), with all differences in qualitative parameters (dendritic orientation and axonal trajectory) being imposed on these neurons by the largely deviant layout of the reeler cortex. Since a major defect in reeler cortical development is the lacking split of the preplate and the subsequently missing clear-cut establishment of a marginal zone and a subplate, these “organizers” of the cortical structure (Kanold and Luhmann, 2010; Genescu and Garel, 2021) are incapable in guiding the ingrowing fibers with their normal radial trajectories. This is best exemplified by the thalamic axons invading the cortical plate in a strongly oblique and recursive manner (Molnar et al., 1998). In such a scenario, the deviations of dendritic orientation in reeler neurons could be an adaptation to maintain an optimal sampling of input from different sources of inputs that have acquired aberrant trajectories.

This dendritic reorientation, together with the loss of layers, would also explain the observed deviant axonal trajectories. Since LVb-fated pyramidal cells contribute with their local recurrent collaterals to the intracortical circuitry in both, intralaminar vs. translaminar ways (Lefort et al., 2009; Campagnola et al., 2022), these collaterals have to detect the respective target cells at unusual positions, probably necessitating a tortuous growth trajectory. Future studies have to show whether these axons in the reeler cortex indeed connect to the same type of target cells with the same physiological properties, as was recently demonstrated in a comprehensive mapping study for the visual cortex (Campagnola et al., 2022).

## Conclusion

Reeler mutant mice have been instrumental to understand many important cellular processes that lead to the formation and maturation of the cortex (Caviness et al., 1988; Tissir and Goffinet, 2003; Frotscher, 2010; Valiente and Marin, 2010). However, the fine-grained analysis of the cellular and synaptic structure of this cortex has been lagging. Since reeler mice show surprisingly subtle disturbance of sensory functions and can use this information for goal-directed behavior, it will be very important to understand what the adaptations of the cortical circuits are to ensure largely proper functioning (Guy and Staiger, 2017).

## Data availability statement

The raw data supporting the conclusions of this article will be made available by the authors, without undue reservation.

## Ethics statement

The animal study was reviewed and approved by Niedersächsisches Landesamt für Verbraucherschutz und Lebensmittelsicherheit.

## Author contributions

JS conceived the research. AS performed all reconstructions. MW analyzed the electrophysiological data. MM analyzed the morphological data. JG complemented this analysis. JS wrote the manuscript with input from all coauthors. All authors contributed to the article and approved the submitted version.

## Funding

This work was funded by the Deutsche Forschungsgemeinschaft through Sta 431/11-2.

## Conflict of interest

The authors declare that the research was conducted in the absence of any commercial or financial relationships that could be construed as a potential conflict of interest.

## Publisher's note

All claims expressed in this article are solely those of the authors and do not necessarily represent those of their affiliated organizations, or those of the publisher, the editors and the reviewers. Any product that may be evaluated in this article, or claim that may be made by its manufacturer, is not guaranteed or endorsed by the publisher.
